# Efficacy of chemical disinfectants for the containment of the salamander chytrid fungus *Batrachochytrium salamandrivorans*

**DOI:** 10.1371/journal.pone.0186269

**Published:** 2017-10-12

**Authors:** Pascale Van Rooij, Frank Pasmans, Yanaika Coen, An Martel

**Affiliations:** Department of Pathology, Bacteriology and Avian Diseases, Faculty of Veterinary Medicine, Ghent University, Merelbeke, Belgium; University of South Dakota, UNITED STATES

## Abstract

The recently emerged chytrid fungus *Batrachochytrium salamandrivorans* (*Bsal*) causes European salamander declines. Proper hygiene protocols including disinfection procedures are crucial to prevent disease transmission. Here, the efficacy of chemical disinfectants in killing *Bsal* was evaluated. At all tested conditions, Biocidal^®^, Chloramine-T^®^, Dettol medical^®^, Disolol^®^, ethanol, F10^®^, Hibiscrub^®^, potassium permanganate, Safe4^®^, sodium hypochlorite, and Virkon S^®^, were effective at killing *Bsal*. Concentrations of 5% sodium chloride or lower, 0.01% peracetic acid and 0.001–1% copper sulphate were inactive against *Bsal*. None of the conditions tested for hydrogen peroxide affected *Bsal* viability, while it did kill *Batrachochytrium dendrobatidis* (*Bd*). For *Bsal*, enzymatic breakdown of hydrogen peroxide by catalases and specific morphological features (clustering of sporangia, development of new sporangia within the original sporangium), were identified as fungal factors altering susceptibility to several of the disinfectants tested. Based on the *in vitro* results we recommend 1% Virkon S^®^, 4% sodium hypochlorite and 70% ethanol for disinfecting equipment in the field, lab or captive setting, with a minimal contact time of 5 minutes for 1% Virkon S^®^ and 1 minute for the latter disinfectants. These conditions not only efficiently target *Bsal*, but also *Bd* and *Ranavirus*.

## Introduction

Skin infection caused by the chytrid fungus *Batrachochytrium salamandrivorans* (*Bsal*) is an important threat to native European salamander species [1,2]. Since its emergence in 2010, *Bsal* has been detected in the Netherlands, Belgium and Germany and will most likely establish permanently within Western Europe [3]. Also the number of captive collections coping with *Bsal* outbreaks is increasing [4–6]. In this context, taking measures to prevent or minimize human-mediated spread of *Bsal* to naïve populations or regions is of paramount importance. The development of proper hygiene protocols, for use in the field, captive collections or laboratories is herein crucial.

For the chytrid fungus *Batrachochytrium dendrobatidis* (*Bd*) and *Ranavirus* (*RV*), key players in global amphibian declines [7–9], a range of effective disinfectants is available. Soaking potentially contaminated equipment in 3–5% sodium hypochlorite (NaOCl, the active ingredient in household bleach), 1% Virkon S^®^ or 70% ethanol (EtOH) for 1 minute is sufficient to inactivate *Bd* [10–12] and *RV* [13,14]. For *Bsal*, however, disinfectant efficacy-studies are non-existing. Following *Bsal* outbreaks in the Netherlands, a hygiene protocol for field-workers using 1% Virkon S^®^ was issued (see e.g. [15]), without prior testing of effectiveness.

The urgent need for hygiene protocols that prevent transmission of *Bsal* in captive and free-ranging environments, prompted us to investigate the efficacy of commonly used chemical disinfectants. Disinfectants with H_2_O_2_ and/or peracetic acid as main active ingredient were included as these might provide a more environmentally friendly alternative (see e.g. [16]) for bleach and Virkon S^®^ [17,18]. For various disinfectants, concentrations and contact times required for 100% killing of *Bsal* were determined *in vitro* and compared to data available for *Bd*. Ideally, the disinfectant of choice should act rapidly and target a broad spectrum of amphibian pathogens.

## Material & methods

### *In vitro* killing of *Bd* and *Bsal* by chemical disinfectants

**Fig 1:**
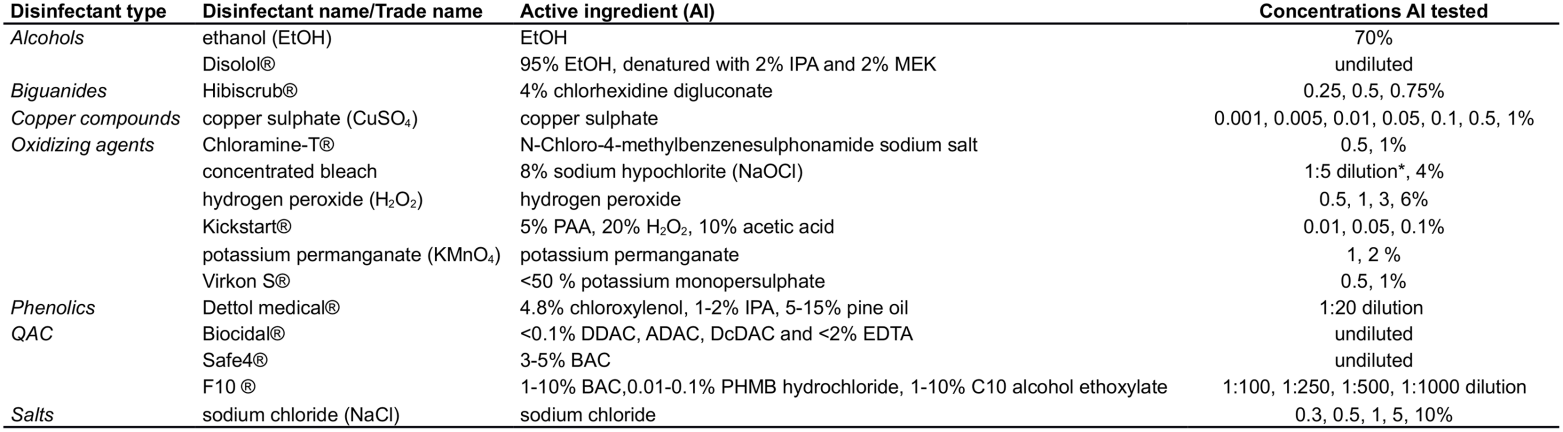
Overview of the disinfectants and the concentration of their active ingredients (AI) tested. ADAC: C12-C16-alkyl dimethyl ammonium chloride, BAC: benzalkonium chloride, DDAC: didecyl dimethyl ammonium chloride, DcDAC: dicoco dimethyl ammonium chloride, EDTA: ethylenediaminetetraacetic acid, IPA: isopropyl alcohol, MEK: methyl ethyl ketone, PAA: peracetic acid, PHMB: polyhexamethylene biguanide, QAC: quaternary ammonium compounds; *1/5 dilution or 1.6% NaOCl: recommended dilution for household purposes.

The fungicidal effect of ethanol (EtOH; VWR, Leuven, Belgium), Disolol^®^ (Chem-lab, Zedelgem, Belgium), Hibiscrub^®^(Regent Medical Ltd., Oldham, UK), copper (II) sulphate (CuSO_4_; Sigma Aldrich, Diegem, Belgium), chloramine-T^®^ (Fagron, Waregem, Belgium), concentrated bleach or sodium hypochlorite (8% NaOCl; Colruyt, Halle, Belgium), hydrogen peroxide (30% H_2_O_2_, stabilized; VWR), Kickstart^®^ (CID-lines, Ieper, Belgium), potassium permanganate (KMnO_4_; VWR), Virkon S^®^ (DuPont, Biosecurity, Ieper, Belgium), Dettol medical^®^ (Reckitt Benckiser, Anderlecht, Belgium), Biocidal^®^ (WAK-Chemie, Steinbach, Germany), Safe4^®^ disinfectant cleaner (diluted spray) (Safe Solutions, Cheshire, UK), F10^®^SC Veterinary disinfectant (Meadows Animal Health, Loughborough, UK) and sodium chloride (NaCl; VWR) on *Bd* and *Bsal* was determined *in vitro* by application of the disinfectants to fungal monolayers as done previously by Berger et al. [19], Johnson et al., [10] and Webb et al. [11], with minor modifications. Concentrations known from literature to be effective against *Bd* or the concentrations recommended by the manufacturer were used as a starting point. An overview of all tested disinfectants with their active components and the concentrations tested is presented in Fig 1. Each disinfectant was diluted to the desired concentration in sterile distilled water. *Bsal* isolate AMFP 13/1 and *Bd* isolate JEL 423 were maintained in TGhL-broth at 15° and 20°C, respectively, following routine methods [1,20]. Zoospores were collected from sporulating broth cultures and diluted in TGhL-broth to a concentration of 2 x 10^5^ zoospores /ml. To each well of a 24-well plate 500 μl zoospore solution was added. Plates were sealed with parafilm and incubated at 15°C (*Bsal*) or 20°C (*Bd*) until semi-confluent monolayers, containing a mixture of all life stages, were obtained. To determine exposure times and concentrations required to kill *Bsal* and *Bd*, the broth was removed and 200 μl of the respective disinfectant was applied onto the monolayers. Sterile distilled water was added to the control wells. For each disinfectant, the monolayers were exposed to the respective disinfectants for 30s, 1, 2, 5 and 10 minutes. At the end of the timed exposure period, the disinfectant was removed. Each well was washed 3 times in 200 μl fresh broth and a final volume of 500 μl fresh broth was added. Plates were sealed, incubated at 15°C (*Bsal*) or 20°C (*Bd*) for 14 days. Plates were examined for growth and the presence of motile zoospores on an inverted microscope at 4, 7, 10 and 14 days after treatment. Each experiment was carried out in triplicate and on 3 independent occasions. Only treatments resulting in 100% kill of all replicates, over all independent repeats were considered effective.

### Catalase assay

Quantification of the cellular catalase activity in *Bd* and *Bsal* isolates was carried out following the protocol of Iwase et al. [21]. A mixture of zoospores and zoosporangia was obtained by flooding 6-day-old broth cultures of *Bd* isolate JEL 423 and *Bsal* isolate AMFP 13/1 (both at passage 13) with sterile distilled water. Cell suspensions were centrifuged for 5 minutes at 2500 rpm, rinsed in sterile distilled water, centrifuged and suspended to a final concentration of 10 mg/100 μl (≈ 1x10^7^ cells/ml). Commercial catalase (from bovine liver, Sigma Aldrich) was dissolved in ultra-pure distilled water and diluted to obtain catalase standards containing 0 to 200 catalase units. Hundred μl of each catalase standard was mixed with 100 μl of a 1% Triton^™^X-100 solution (Sigma Aldrich) and 100 μl of a 30% H_2_O_2_ solution, in a borosilicate reagent tube (13mm x 10mm, VWR). Hundred μl of the *Bd* or *Bsal* cell suspensions was mixed with equal volumes of Triton^™^X-100 and H_2_O_2_. Samples were incubated at room temperature for 15 min. During incubation, present catalases break H_2_O_2_ down into H_2_O and O_2_; O_2_-bubbles are trapped by the surfactant Triton^™^X-100 and visualized as a foam column in the test tube. The height of the foam column in each reagent tube was measured with a ruler. The catalase activity for each sample was calculated from the standard curve obtained by plotting the defined units of catalase activity. Mean catalase activity was measured in three independent assays.

### *In vitro* susceptibility of *Bsal* life stages and infectious propagules

The effect of 1% Virkon^®^ on motile *Bsal* zoospores, encysted spores and zoosporangia was compared as followed. (1) Encysted *Bsal* spores were collected from a 10 days old sporulating culture by carefully applying a sterile microbiological loop (Microloop, 10 μl, Biosigma S.r.l. Cona, Italy) onto the liquid surface of the culture. Five loopfuls of encysted spores, corresponding with a total of 50 μl, were taken out and transferred into a sterile tube containing 400 μl broth by twirling around the loop in the broth. (2) For the collection of motile zoospores, 5-day old broth cultures were provided with sterile distilled water for 24 hours or until zoospores were released from the mature sporangia. Zoospore suspensions were collected and filtered over a 10 μm mesh cell strainer (pluristrainer, pluriSelect Life Sciences, Leipzig, Germany) to remove sporangia from the spore suspension. (3) Immature sporangia were collected from non-sporulation broth-cultures as following: *Bsal* monolayers were first rinsed with TGhL-broth, and then collected by gently scraping the sporangia from the cell culture flasks using a cell scraper. Spores and sporangia were counted in lugol using a haemocytometer and adjusted to 1 x10^6^ spores or sporangia/ 400 μl. Samples were centrifuged for 1 min. at 2500 rpm. The supernatant was removed; the pellets in the test tubes were dissolved in 200 μl 1% Virkon^®^ and incubated for 1 minute, whereas the pellets in the control tubes were dissolved in sterile distilled water. After incubation 200 μl broth was added to reduce the disinfectant action. The disinfectant was removed by centrifugation (1 min at 2500 rpm) and the pellet was washed 3 times in broth. Finally, the pellet was suspended in 400 μl broth, transferred into a 48-well plate, sealed, incubated at 15°C and observed after 4, 7, 10, 14 and 20 days.

## Results

### *In vitro* killing of *Bd* and *Bsal* cultures by chemical disinfectants

**Fig 2:**
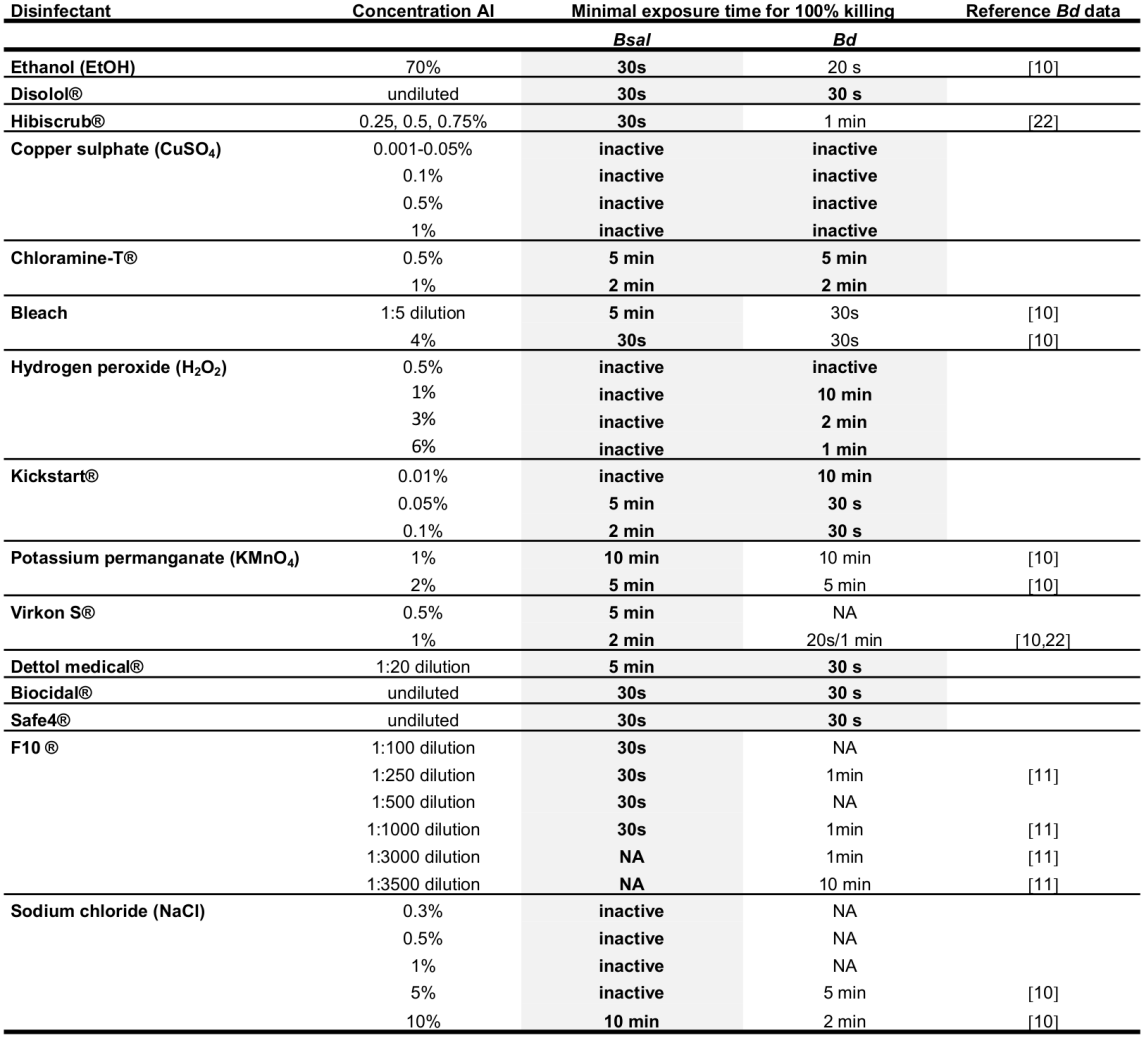
Efficacy of chemical disinfectants in killing *Bsal* and *Bd*. Fig 2 summarises the effect of various chemical disinfectants on zoospores and zoosporangia of *Bsal* and *Bd* after exposure to listed concentrations and contact times. AI: active ingredient. Conditions shaded grey were evaluated *in vitro* during this study while other data were gathered from literature.

Effective disinfectant concentrations and required contact times to achieve 100% killing of *Bd* and *Bsal* cultures are summarized in Fig 2. All concentrations tested for Biocidal^®^, Safe4^®^, F10^®^, 70% EtOH, Disolol^®^and Hibiscrub^®^ and bleach containing 4% sodium hypochlorite (NaOCl) displayed a rapid action and killed *Bd* and *Bsal* within 30 seconds. For Chloramine-T^®^, contact times of respectively 5 minutes (0.5% dilution) and 2 minutes (1% dilution) were required for complete killing of the cultures, whereas for KMnO_4_ 10 minutes (1% dilution) and 5 minutes (2% dilution) minutes were required.

Several disinfectants were less active against *Bsal* than against *Bd*. These disinfectants include Dettol medical^®^, concentrated bleach at a 1:5 dilution (≈ 1.6% NaOCl) in water which is recommended for household use, 1% Virkon S^®^, 10% salt (NaCl) and Kickstart^®^ dilutions containing 0.05–0.1% peracetic acid (PAA). In general, these disinfectant concentrations killed *Bd* within 30 seconds, while for *Bsal* longer contact times (up to 5 minutes) were required. For 10% NaCl, 2 and 10 minutes were required to kill *Bd* and *Bsal* respectively.

Kickstart^®^ dilutions containing 0.01% PAA and 5% NaCl dilutions, effective against *Bd*, were ineffective against *Bsal*. *Bd* was also found more susceptible to inactivation by H_2_O_2_ than *Bsal*. *Bd* was resistant to 0.5% H_2_O_2,_ but was killed by 1–6% H_2_O_2_. In contrast, *Bsal* killing could only be achieved by application of a 6% H_2_O_2_ dilution for at least 15 minutes. The efficacy of H_2_O_2_ at concentrations of 10% and above was not tested because of their strongly irritating and corrosive nature. Application of 3–6% H_2_O_2_ onto *Bsal* monolayers provoked an effervescent reaction. This was not observed for *Bd*. CuSO_4_ did not kill *Bd* or *Bsal*, at either concentration tested.

On several occasions, clear growth of *Bsal* sporangia into mature zoosporangia and/or the presence of motile zoospores only occurred 10–14 days after the initial disinfectant treatment. A summary of the respective conditions under which this occurred is given in Table 1. The sporangia were typically non-sessile and were clustered with several other sporangia. This was not observed in the controls treated with water.

**Table 1:** Suboptimal disinfectant conditions for *Bsal*.

Disinfectant concentration	Exposure time
Concentrated bleach, 1:5 dilution	2 min.
Dettol medical^®^, 1:20 dilution	30 s., 1–2 min.
H_2_O_2_, 1%	10 min.
H_2_O_2_, 6%	10 min.
Kickstart^®^, 0.05% PAA	2 min.
Kickstart^®^, 0.1% PAA	30 s.
KMnO_4,_ 2%	5 min.
KMnO_4,_ 1%	2 min.
Virkon S^®^, 0.5%	1 min.
Virkon S^®^, 1%	1 min.

For all listed conditions, fungal growth reoccurred 10–14 days after the initial treatment. H_2_O_2_: hydrogen peroxide; PAA: peracetic acid; KMnO_4_: potassium permanganate.

### Catalase assay

**Fig 3:**
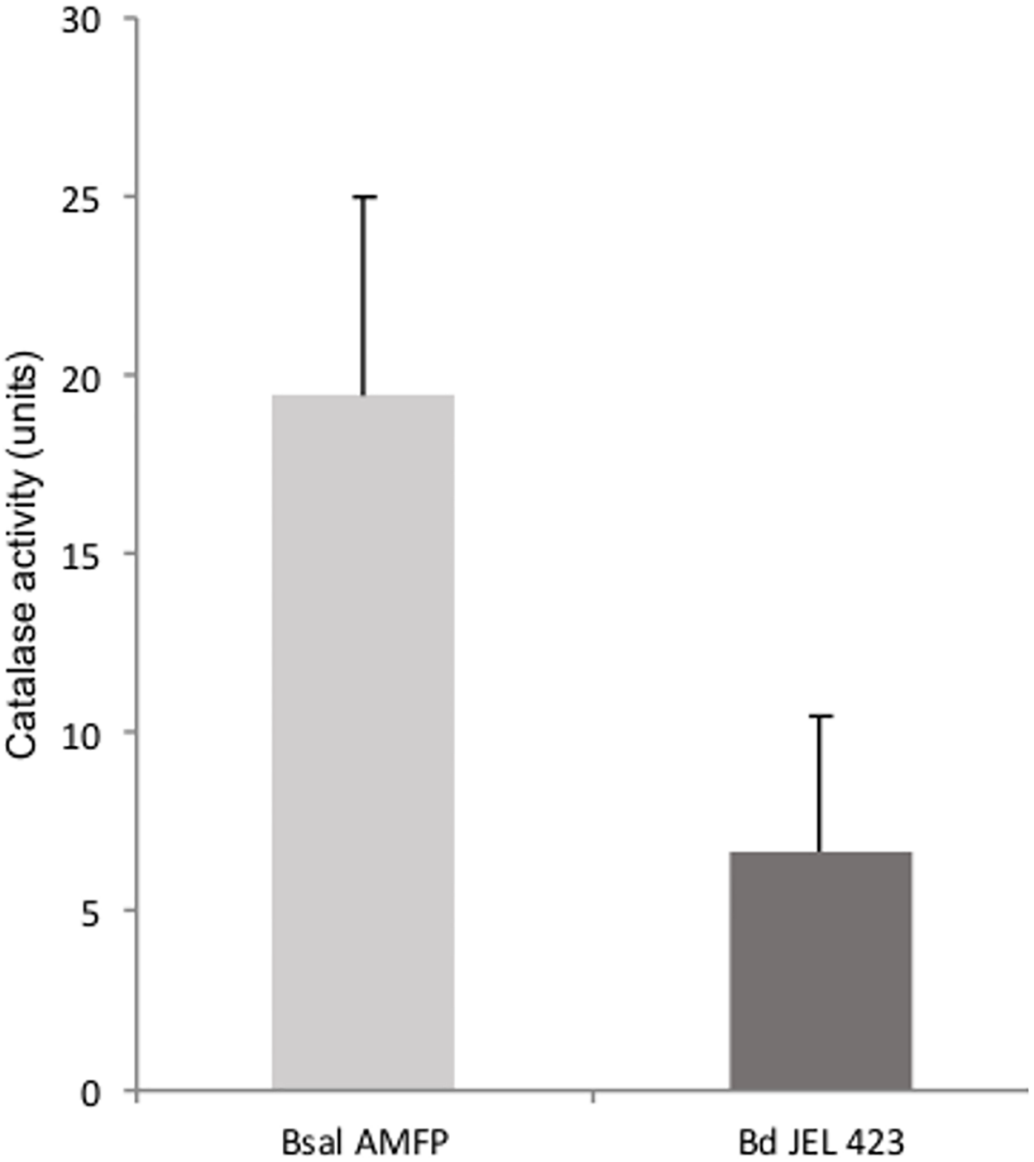
Catalase activity of *Bsal* isolate AMFP and *Bd* isolate JEL 423. Mean values are shown (n = 3). Error bars represent the standard deviation (SD).

The production of bubbles following exposure of *Bsal* monolayers to H_2_O_2_ was indicative of catalase activity. Catalase enzymes break down H_2_O_2_ into oxygen (visible as air bubbles) and water and may explain the increased tolerance of *Bsal* against H_2_O_2_. Cellular catalase activity in *Bd* and *Bsal* isolates was measured. The catalase activity of *Bsal* cells was 2.3 to 4 higher than that of *Bd* cells (S1 Table). For *Bsal* a mean catalase activity ± standard deviation (SD) of 19.43 ± 5.56 units (U) was found compared to 6.68 ± 3.79 U for *Bd* (Fig 3 and S1 Table).

### *In vitro* susceptibility of *Bsal* life stages and infectious propagules

To compare the susceptibility of several *Bsal* life stages, samples containing (1) encysted *Bsal* spores only, or (2) motile zoospores or (3) immature sporangia were exposed to 1% Virkon S^®^ for 1 minute. Exposure of encysted and motile spores to Virkon S^®^ resulted in complete killing.

Over the 14-day period following the treatment with Virkon S^®^, the exposed immature sporangia developed into non-sessile sporangia clustering together. Each sporangium within the cluster did not release motile zoospores. Instead, each sporangium produced immotile spores that developed into new immature sporangia within the original sporangium. In the control samples, the immature sporangia developed into mature sporangia releasing motile zoospores.

## Discussion

Our results show that a number of chemical disinfectants cause 100% mortality of *Bsal in vitro* within a relatively short time. Biguanide disinfectants containing 0.75% chlorhexidine such as Hibiscrub^®^ or Nolvasan^®^, bleach containing 4% NaOCl and 70% EtOH kill *Ranavirus*, *Bd* and *Bsal in vitro* within 1 minute [10–14, 22]. Also 1% Virkon S^®^ is active against all 3 amphibian pathogens but a minimal contact time of 5 minutes needs to be respected to destroy *Bsal* [10,14,22]. The results of this study also highlight the efficacy of the quaternary ammonium compounds (QACs) F10 SC^®^, Biocidal^®^ and Safe 4^®^ against chytrid fungi. QACs are surfactants and are good cleaning agents due to their detergent properties [23]. They are fast acting and active at very low concentrations (1:1000–1:6400) which reduces costs (see e.g. [10,11,24]) and environmental toxicity. Although QACs are not recommended for disinfecting instruments, they may be useful for disinfection of footwear, collection equipment or containers [10]. It is important to note that QACs are not effective against several non-enveloped viruses, several pathogenic fungi, bacterial spores and mycobacteria [23,25]. Literature data on the efficacy of QACs against ranaviruses, that may occur as both non-enveloped and enveloped particles [26], are lacking.

For selecting the disinfectant of choice, residual activity, effects on fabric and metal, toxicity to the environment and aquatic organisms, relative safety to people, associated costs, availability and possible inactivation of the disinfectant in presence of organic matter and other compounds such as soap, should be considered. Although bleach is highly toxic to aquatic organisms [17] and corrosive, it is relatively fast acting, inexpensive, unaffected by water hardness [25]. Moreover, it is one of the sole disinfectants that is widely available, also for hobbyists. Alcohols are also fast acting but are too expensive for general use. They are most effective when diluted with distilled water to 60–90% as this facilitates diffusion through the pathogen’s cell membrane, while 100% alcohol only denatures external membrane proteins [23]. The use of denatured alcohols (alcohols to which isopropyl alcohol and methyl ethyl ketone is added) may provide a considerably cheaper alternative for disinfection of instruments (scissors, callipers, balances) and hard surfaces (benches).

Second, the conditions required to fully inactivate *Bsal* (and other amphibian EIDs) in the field may differ from those under controlled laboratory conditions. (1) Sufficient time and the appropriate temperature must be allowed for action of the disinfectant and may depend on the degree of contamination and organic matter load [25]. The efficacy of e.g. bleach, Chloramine-T^®^ and chlorhexidine-based disinfectants (such as Hibiscrub^®^ and Nolvasan^®^) is compromised by organic matter and thus it is important to remove mud, soil, plant material form the equipment or boots prior to disinfection [23,25]. (2) Furthermore, solutions for disinfection should be used according to the manufacturer’s instructions to ensure adequate levels of their active ingredient(s) for microbial activity. In particular when using bleach, always verify the NaOCl concentrations on the labels of individual containers; ‘concentrated’ bleach contains 8% NaOCl, while ‘regular’ bleach only contains 5% NaOCl. Be aware that older bottles of disinfectants are likely to have lower concentrations of active ingredient(s), in order to avoid overly dilute attempts at sanitizing. Chlorine based disinfectants (e.g. bleach, Chloramine-T^®^) diluted in tap water and stored at room temperature have a limited shelf life [25,27]. (3) Finally, it is crucial that field equipment that has been in contact with amphibians, pond water or mud, such as boots and nets etc., are rigorously disinfected and left to dry between use and before moving to another field site. Equipment should be soaked in a disinfectant bath allowing the disinfectants to act onto potential infectious propagules during the prescribed contact time.

Compared to *Bd*, several disinfectants appeared less active or even inactive against *Bsal*. The fungal cell wall presents an important barrier against antifungal agents [28]. There are only few studies tackling this subject and most of them concern resistance mechanisms in yeasts. In these latter, cell wall composition (mainly elevated levels of β-1,3 glucan), wall thickness, and relative porosity are linked to antifungal susceptibility (see e.g. [23, 29]). Chytrid zoospores are surrounded by a plasma membrane, but whenever a zoospore ‘settles’, or retracts its flagellum and encysts, a cell wall is formed which increases in size and thickness to become a sporangium [20,30]. The cell wall may act as a permeability barrier, excluding or reducing the uptake of a disinfectant [23]. Disinfectants such as CuSO_4,_ that affect zoospore viability through absorption by the cell membrane and subsequent metabolic inhibition [31,32] do not affect *Bd* or *Bsal* sporangia. Disinfectants such as KMnO_4_ and Chloramine-T^®^ (damaging the cell membrane by oxidation of cell membrane associated molecules, leading to increased cell permeability) or NaCl (affecting cellular membrane integrity by osmotic stress)[23], have a selective disadvantage against sporangia and thus higher disinfectant concentrations are required to inhibit sporangia than for zoospores. This has also been observed for *Bd* [19]. Unique to *Bsal* is the production of encysted spores inside sporangia [33]. The cell wall of the zoosporangium and the encysted spores within it, provide a double barrier against the action of the disinfectants. This may explain (partially) why higher disinfectant concentrations or a longer contact time are necessary to achieve full fungal killing of *Bsal*, compared to those necessary for inhibition of *Bd*. Also the clustering of multiple *Bsal* zoosporangia may protect centrally located sporangia from the full impact of a given disinfectants.

Enzymatic degradation of antimicrobial agents by means of catalases or peroxidases is a common strategy in pathogenic bacteria [34,35] and fungi [36,37]. Catalases are able to ‘detoxify’ H_2_O_2_ by breakdown into H_2_O and O_2_, which is visualised by a bubbling reaction. The presence of catalases can increase tolerance to low concentrations of H_2_O_2_ and thus higher concentrations of H_2_O_2_ (10–30%) or longer contact times are required for antimicrobial activity [23]. The catalase activity of the *Bsal* isolate used for the *in vitro* killing assays, was approximately 3 times higher than in *Bd*. The increased abundance of catalases in *Bsal* may explain why *Bsal* is resistant to H_2_O_2_ and less susceptible to Kickstart^®^ (which has H_2_O_2_ as main active ingredient; see Fig 1) than *Bd*. The presence of catalases in *Bsal* and their role in resistance against H_2_O_2_ are novel observations. For *Bd*, catalase genes had already been described from its genome but until now catalase action had not yet been quantified *in vitro* [38,39]. Although not yet explored, the pronounced catalase activity in *Bsal* may have implications for the host’s immune defences. Several pathogenic bacteria and fungi use catalases to neutralise the H_2_O_2_ coming from phagocytes [34–37]. Evasion of defences allows these pathogens to survive in inflammatory foci (see e.g. [34]). Further research is necessary to shed more light on the role of catalases in the pathogenesis and virulence of *Bsal*.

Proper hygiene protocols including appropriate disinfection procedures are of utmost importance to contain human-mediated spread of *Bsal* into naïve populations or regions. Based on the *in vitro* results, we recommend 1%Virkon S^®^, 4% NaOCl and 70% EtOH for disinfecting equipment for use in the field, laboratory and captive husbandry, with a minimal contact time of 5 minutes to be respected for 1%Virkon S^®^, and 1 minute for the latter disinfectants. These conditions not only efficiently target *Bsal*, but also *Bd* and *Ranavirus*.

## Supporting information

S1 Table Raw data of the catalase assays. SD: standard deviation.
(PDF)

## References

[1] Martel A, Spitzen-van der Sluijs A, Blooi M, Bert W, Ducatelle R, Fisher MC, et al *Batrachochytrium salamandrivorans* sp. nov. causes lethal chytridiomycosis in amphibians. Proc Natl Acad Sci USA. 2013;110: 15325–15329. doi: 10.1073/pnas.1307356110 24003137 10.1073/pnas.1307356110 PMC3780879

[2] Martel A, Blooi M, Adriaensen C, Van Rooij P, Beukema W, Fisher MC, et al Recent introduction of a chytrid fungus endangers Western Palearctic salamanders. Science. 2014;346: 630–1 doi: 10.1126/science.1258268 25359973 10.1126/science.1258268 PMC5769814

[3] Spitzen-van der Sluijs A, Martel A, Asselberghs J, Bales EK, Beukema W, Bletz MC, et al Expanding distribution of lethal amphibian fungus *Batrachochytrium salamandrivorans* in Europe. Emerg Infect Dis. 2016;22: 1286 doi: 10.3201/eid2207.160109 27070102 10.3201/eid2207.160109 PMC4918153

[4] Cunningham AA, Beckmann K, Perkins M, Fitzpatrick L, Cromie R, Redbond J, O'Brien MF, Ghosh P, Shelton J, Fisher MC. Emerging disease in UK amphibians. Vet Rec. 2015;176: 468. 10.1136/vr.h2264 25934745

[5] Sabino-Pinto J, Bletz M, Hendrix R, Bina Perl RG, Martel A, Pasmans F, et al First detection of the emerging fungal pathogen *Batrachochytrium salamandrivorans* in Germany. Amphib-Reptil. 2015;36: 411–416.

[6] Fitzpatrick L, Pasmans F, Martel A, Cunningham A. Epidemiological tracing of Batrachochytrium salamandrivorans in European private amphibian collection. In Proc. In: Schumann A, Wibbelt G, Greenwood AD, Hofer H, editors. Proceedings of the 12th conference of the European Wildlife Disease Association (EWDA); 2016 Aug 26–31; Berlin, Germany. Berlin: Leibniz Institute for Zoo and Wildlife Research, 2016. p. 30.

[7] Van Rooij P, Martel A, Haesebrouck F, Pasmans F. Amphibian chytridiomycosis: a review with focus on fungus-host interactions. Vet Res. 2015;46: 137 doi: 10.1186/s13567-015-0266-0 26607488 10.1186/s13567-015-0266-0 PMC4660679

[8] Green DE, Converse KA, Schrader AK Epizootiology of sixty-four amphibian morbidity and mortality events in the USA, 1996–2001. Ann NY Acad Sci. 2002; 969: 323–333. 12381613 10.1111/j.1749-6632.2002.tb04400.x

[9] Duffus ALJ, Waltzek TB, Stöhr AC, Allender MC, Gotesman M, Whittington RJ, et al Distribution and host range of ranaviruses In: Gray MJ, Chinchar VG, editors. Ranaviruses: Lethal pathogens of ectothermic vertebrates. Heidelberg: Springer; 2015 p. 9–58.

[10] Johnson ML, Berger L, Philips L, Speare R. Fungicidal effects of chemical disinfectants, UV light, desiccation and heat on the amphibian chytrid *Batrachochytrium dendrobatidis*. Dis Aquat Organ. 2003;57: 255–260. doi: 10.3354/dao057255 14960039 10.3354/dao057255

[11] Webb R, Mendez D, Berger L, Speare R. Additional disinfectants effective against the amphibian chytrid fungus *Batrachochytrium dendrobatidis*. Dis Aquat Organ. 2007; 74: 13–6. doi: 10.3354/dao074013 17425259 10.3354/dao074013

[12] Phillott AD, Spear R, Hines HB, Meyer E, Skerratt LF, McDonald KR, et al Minimizing exposure of amphibians to pathogens during field studies. Dis Aquat Organ. 2010;92: 175‐185. doi: 10.3354/dao02162 21268979 10.3354/dao02162

[13] Langdon JS. Experimental transmission and pathogenicity of epizootic haematopoietic necrosis virus (EHNV) in redfin perch, *Perca fluviatilis* L., and 11 other teleosts. J Fish Dis. 1989;12: 295–310.

[14] Bryan LK, Baldwin CA, Gray MJ, Miller DL. Efficacy of select disinfectants at inactivating *Ranavirus*. Dis Aquat Organ. 2009;84: 89–94. doi: 10.3354/dao02036 19476278 10.3354/dao02036

[15] RAVON Hygiene protocol [Internet]. Nijmegen: Reptielen Amfibieën Vissen Onderzoek Nederland; c2017 [cited 2017 April 20]. http://www.ravon.nl/Infotheek/Protocollen/Hygieneprotocol/tabid/1513/Default.aspx.

[16] De Swaef E, Van den Broeck W, Dierckens K, Decostere A. Disinfection of teleost eggs: a review. Rev Aquacult. 2015;7: 1–21.

[17] Schmidt BR, Geiser C, Peyer N, Keller N, von Rütte M. Assessing whether disinfectants against the fungus *Batrachochytrium dendrobatidis* have negative effects on tadpoles and zooplankton. Amphib-Reptil. 2009;30: 313–319.

[18] Hangartner S, Laurila A. Effects of the disinfectant Virkon S on early life-stages of the moor frog (*Rana arvalis*). Amphib-Reptil. 2012;33: 349–353.

[19] Berger L, Speare R, Pessier A, Voyles J, Skerratt LF. Treatment of chytridiomycosis requires urgent clinical trials. Dis Aquat Organ. 2010;92: 165–174. doi: 10.3354/dao02238 21268978 10.3354/dao02238

[20] Longcore JE, Pessier AP, Nichols DK. *Batrachochytrium dendrobatidis* gen et sp nov, a chytrid pathogenic to amphibians. Mycologia. 1999;91: 219–227.

[21] Iwase T, Tajima A, Sugimoto S, Okuda K, Hironaka I, Kamata Y, et al A simple assay for measuring catalase activity: a visual approach. Sci Rep. 2013;3: 3081 doi: 10.1038/srep03081 24170119 10.1038/srep03081 PMC3812649

[22] Gold KK, Reed PD, Bemis DA, Miller DL, Gray MJ, Souza MJ, et al Efficacy of common disinfectants and terbinafine in inactivating the growth of *Batrachochytrium dendrobatidis* in culture. Dis Aquat Organ. 2013;107: 77–81. doi: 10.3354/dao02670 24270026 10.3354/dao02670

[23] McDonnell G, Russell AD. Antiseptics and disinfectants: activity, action and resistance. Clin Microbiol Rev. 1999;12: 147–179. 9880479 10.1128/cmr.12.1.147 PMC88911

[24] Blooi M, Martel A, Vercammen F, Pasmans F. Combining ethidium monoazide treatment with real-time PCR selectively quantifies viable *Batrachochytrium* cells. Fungal Biol. 2013;117: 156–162. doi: 10.1016/j.funbio.2013.01.004 23452953 10.1016/j.funbio.2013.01.004

[25] CDC Guideline for Disinfection and Sterilization in Healthcare Facilities [Internet]. Atlanta: Centre for Disease Control; c2008 [cited 2017 April 20]. https://www.cdc.gov/hai/pdfs/disinfection_nov_2008.pdf

[26] Chinchar VG, Yu KH, Jancovich JK. The molecular biology of Frog Virus 3 and other Iridoviruses infecting cold-blooded vertebrates. Viruses. 2011;3: 1959–1985. doi: 10.3390/v3101959 22069524 10.3390/v3101959 PMC3205390

[27] Clarkson RM, Moule AJ, Podlich HM. The shelf-life of sodium hypochlorite irrigating solutions. Aust Dent J. 2001;46: 269–76. 11838874 10.1111/j.1834-7819.2001.tb00291.x

[28] Sanglard D, Odds F. Resistance of *Candida* species to antifungal agents: molecular mechanisms and clinical consequences. Lancet Infect Dis. 2002;2: 73–85. 11901654 10.1016/s1473-3099(02)00181-0

[29] Mesa-Arango AC, Rueda C, Román E, Quintin J, Terrón MC, Luque D, et al Cell wall changes in amphotericin B-resistant strains from *Candida tropicalis* and relationship with the immune responses elicited by the host. Antimicrob Agents Chemother. 2016;60: 2326–35. doi: 10.1128/AAC.02681-15 26833156 10.1128/AAC.02681-15 PMC4808153

[30] Van Rooij P, Martel A, D'Herde K, Brutyn M, Croubels S, Ducatelle R, et al Germ tube mediated invasion of *Batrachochytrium dendrobatidis* in amphibian skin is host dependent. PLoS One. 2012;7: e41481 doi: 10.1371/journal.pone.0041481 22911798 10.1371/journal.pone.0041481 PMC3401113

[31] Boivert SP, Davidson E.W. Growth of the amphibian pathogen, *Batrachochytrium dendrobatidis*, in response to chemical properties of the aquatic environment. J Wildl Dis. 2011;47: 694–698. doi: 10.7589/0090-3558-47.3.694 21719835 10.7589/0090-3558-47.3.694

[32] Caldwell B, Sideman E, Seaman A, Shelton A, Smart C. Resource guide for organic insect and disease management. 2^nd^ ed New York: New York State Agricultural Experiment Station (NYSAES), Cornell University; 2013.

[33] Stegen G, Pasmans F, Schmidt BR, Rouffaer LO, Van Praet S, Schaub M, et al Drivers of salamander extirpation mediated by *Batrachochytrium salamandrivorans*. Nature. 2017;544: 353–356. doi: 10.1038/nature22059 28425998 10.1038/nature22059

[34] Hassett DJ, Cohen MS. Bacterial adaptation to oxidative stress: implications for pathogenesis and interaction with phagocytic cells. FASEB J. 1989;3: 2574–2582. 2556311 10.1096/fasebj.3.14.2556311

[35] Flannagan RS, Cosío G, Grinstein S. Antimicrobial mechanisms of phagocytes and bacterial evasion strategies. Nature Rev Microbiol. 2009;7: 355–366. 19369951 10.1038/nrmicro2128

[36] Da Silva Dantas A, Day A, Ikeh M, Kos I, Achan B, Quinn J. Oxidative stress responses in the human fungal pathogen, *Candida albicans*. Biomolecules. 2015;5: 142–165. doi: 10.3390/biom5010142 25723552 10.3390/biom5010142 PMC4384116

[37] Singh P, Paul S, Shivaprakash MR, Chakrabarti A, Ghosh AK. Stress response in medically important Mucorales. Mycoses. 2016;59: 628–635. doi: 10.1111/myc.12512 27292160 10.1111/myc.12512

[38] Zamocky M, Gasselhuber B, Furtmüller PG, Obinger C. Turning points in the evolution of peroxidase-catalase superfamily: molecular phylogeny of hybrid heme peroxidases. Cell Mol Life Sci. 2014;71: 4681–4696. doi: 10.1007/s00018-014-1643-y 24846396 10.1007/s00018-014-1643-y PMC4232752

[39] UniProtKB—F4P1R4 (F4P1R4_BATDJ)[Internet]. UniProt Consortium; 2002–2017. Protein sequence and annotation data [cited 2017 Apr 20]. http://www.uniprot.org/uniprot/F4P1R4.

