# Supplementary material for: Efficacy of chemical disinfectants for the containment of the salamander chytrid fungus Batrachochytrium salamandrivorans
Source: PLoS One. 2017 Oct 12;12(10):e0186269. doi: 10.1371/journal.pone.0186269 (PMC5638399; doi:10.1371/journal.pone.0186269)
Supplement: S1 Table — SD: standard deviation. (PDF) [file pone.0186269.s001.pdf]

	Bsal		*Bd*	
Assay	height foam column (mm)	catalase activity (units)	height foam column (mm)	catalase activity (units)
1	12.00	25.50	8.00	10.93
2	9.00	14.57	6.00	3.64
3	10.00	18.21	6.50	5.46
mean ± SD	10.33±1.53	19.43±5.56	6.83±1.04	6.68±3.79
